# Incidental Finding of Persistent Trigeminal Artery in a Young Woman: A Case Report

**DOI:** 10.7759/cureus.61060

**Published:** 2024-05-25

**Authors:** Andrei Manea, Sergiu Ștefan Laszlo, Adina Stoian, Iuliu Gabriel Cocuz, Ioana Halmaciu

**Affiliations:** 1 Department of Radiology, Mureș County Emergency Hospital, Târgu Mureș, ROU; 2 Intensive Care Unit, Mureș County Hospital, Târgu Mureș, ROU; 3 Department of Pathophysiology, George Emil Palade University of Medicine, Pharmacy, Sciences and Technology, Târgu Mureș, ROU; 4 Mures County Clinical Emergency Hospital, 1st Neurology Clinic, Târgu Mureș, ROU; 5 Pathology Department, Mures Clinical County Hospital, Târgu Mureș, ROU; 6 Department of Radiology, George Emil Palade University of Medicine, Pharmacy, Sciences and Technology, Târgu Mureș, ROU; 7 Department of Radiology, Mures County Clinical Emergency Hospital, Târgu Mureș, ROU

**Keywords:** persistent trigeminal artery, carotid-vertebrobasilar anastomoses, incidental radiological finding, brain vascular malformation, magnetic resonance angiography

## Abstract

Persistent trigeminal artery disease is one of the most common types of persistent carotid-vertebrobasilar anastomoses. Usually, it is unilateral, and it can be discovered with a magnetic resonance angiography (MRA), computed tomography angiography (angioCT), or classic angiography exam. It can be associated with non-specific symptoms, such as headaches, or more specific ones, such as III or VI nerve palsy or trigeminal neuralgia, but most of the time it goes undetected, being an incidental finding and not causing any symptoms. On MRA and angioCT, it has the characteristic “tau” sign. We present the case of a young woman who, incidentally, discovered this malformation after undergoing an MRA. She had been experiencing a persistent headache without a known cause, which did not improve despite medication.

## Introduction

When the embryo is about six weeks old, the trigeminal artery forms, connecting the cavernous portion of the developing internal carotid arteries (ICAs) with the paired longitudinal neural arteries (PLNAs) [1]. It then involutes when the posterior communicating artery is done developing, later in the fetal period [2]. The reasons for the persistence of the trigeminal artery (PTA) are unknown, as it is the most common persistent embryonic carotid-basilary anastomosis [3]. It is named the trigeminal artery because of its proximity to the trigeminal ganglion. Classic angiography, computer tomography angiography with contrast substance (angioCT), or magnetic resonance angiography (MRA) can diagnose this malformation, with most diagnoses occurring incidentally during MRA scans. The prevalence varies from 0.061% to 1% using MRA, angioCT, or classic angiography [4-5].

Richard Quinn first reported a PTA during an autopsy in 1844, and Sutton first observed it in a living patient during an angiography in 1950 [6]. The specific sign that can be seen on an MRA or angioCT scan is the “tau” or trident sign, which is best seen on a sagittal view [7].

A 2010 study by O’uchi et al. reports that there is a frequent coexistence of PTA and other cerebrovascular anomalies, which may suggest that errors in early arterial development may cause this malformation [4].

Clinical symptoms associated with its presence include trigeminal neuralgia resulting from vascular compression, ocular pain, and pulsatile exophthalmia caused by a trigeminal cavernous fistula, or nerve palsy resulting from an aneurysm at the level of the affected nerve, typically the III, IV, V, or VI cranial nerves.

Classification

The different variations of the PTA can be cataloged using the Saltzman classification or the Salas classification. Saltzman divides the PTA into three major types (I, II, and III), while type III is subdivided into types III a, b, or c [2-3].

Type I PTA enters the basilar artery (BA) between the superior cerebellar artery (SCA) and anterior inferior cerebellar artery (AICA); in this case, the PTA supplies the BA, SCAs, and posterior cerebral arteries (PCAs), while the posterior communicating arteries (PComAs), vertebral arteries (VAs), and proximal BA can be absent of hypoplastic; this could be confused with stenosis [2-3]. Type I can lead to posterior circulation symptoms if there is a reduction of blood flow in the anterior circulation [8].

Type II PTA is very similar to type I, except for the fact that the proximal BA and PComAs are well formed and contribute to the distal posterior circulation. There is also a combination between types I and II in which one PCA and both SCA and BA are supplied by the PTA, and one PCA is supplied by the PComA [2-3]. A study by Geibparsert et al. from 2008 included 17 patients with cavernous fistulas, and all of them had a type II PTA [9].

Type III PTA terminates as SCA (type IIIa), AICA (type IIIb), or posterior inferior cerebellar artery (PICA). Type IIIb is the common of the type III [2-3].

An easier way to remember the clinical implications of each specific type is to know to what territory each type of PTA goes and the specific symptoms of each territory. Type I supplies the posterior circulation, having hypoplastic BA, VA, and PComAs. Type II supplies the superior cerebellar arteries, while PCSs are supplied by PComAs, resulting in milder symptoms in cases of occlusion than type I. Type III does not interfere with the posterior circulation, terminating as one of the cerebellar arteries [2-3,10].

In one study of 4.650 patients that underwent brain MRA, the prevalence of each type using the Saltzman classification was as follows: type I, 24%; type II, 16%; and type III, 60% [11].

Another classification used is the Salas classification, which categorizes the PTA into lateral and medial types regarding its relationship with the abducens nerve (cranial nerve VI). If the origin of the PTA is the posterolateral aspect of the posterior bend of the ICA (C4 segment), crossing underneath the abducens nerve and continuing between the abducens and trigeminal nerves, this is the lateral type. If the PTA takes a medial course, it arises from the posteromedial aspect of the posterior bend of the ICA, piercing the clival dura at the dorsum sallae [12]. This could be a cause of VI nerve palsy due to its proximity to the nerve [6].

## Case presentation

The patient, a 20-year-old woman with no comorbidities, presented at a private medical center in Târgu Mureș for a brain MRI, accusing persistent headaches that do not ameliorate when using medication. A 3D time of flight (TOF) sequence was also done, discovering a PTA that originated in the posteromedial aspect of the ICA C4 segment, crossing over the dura, having an extradural trajectory lateral to the hypophysis, and inserting between the SCA and AICA. The proximal segment of the BA is hypoplastic, while the PComAs are aplastic. There was no aneurysm associated with the PTA. 

**Figure 1:**
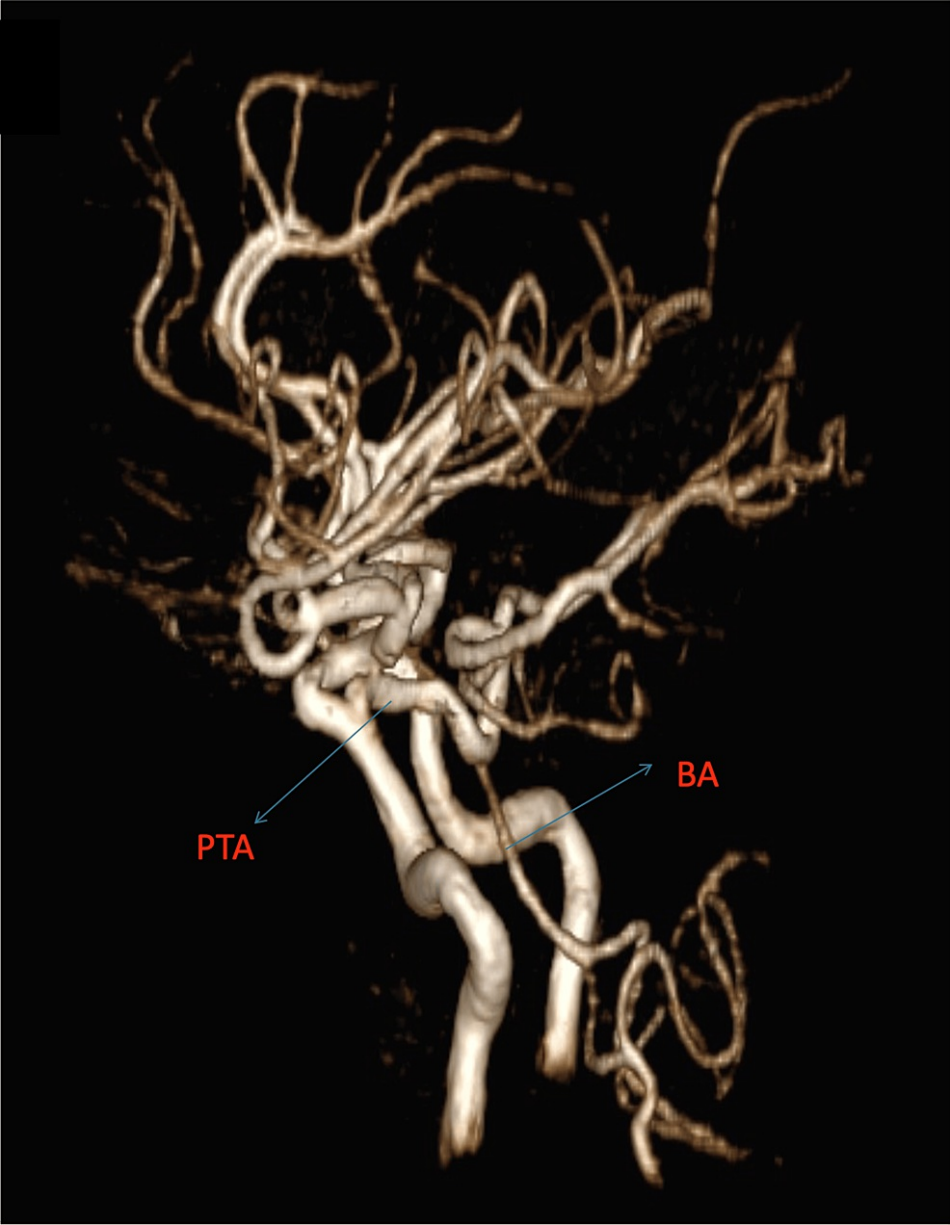
Sagital view of the 3D reconstruction of the 3D TOF sequence PTA: persistent trigeminal artery, BA: basilar artery

**Figure 2:**
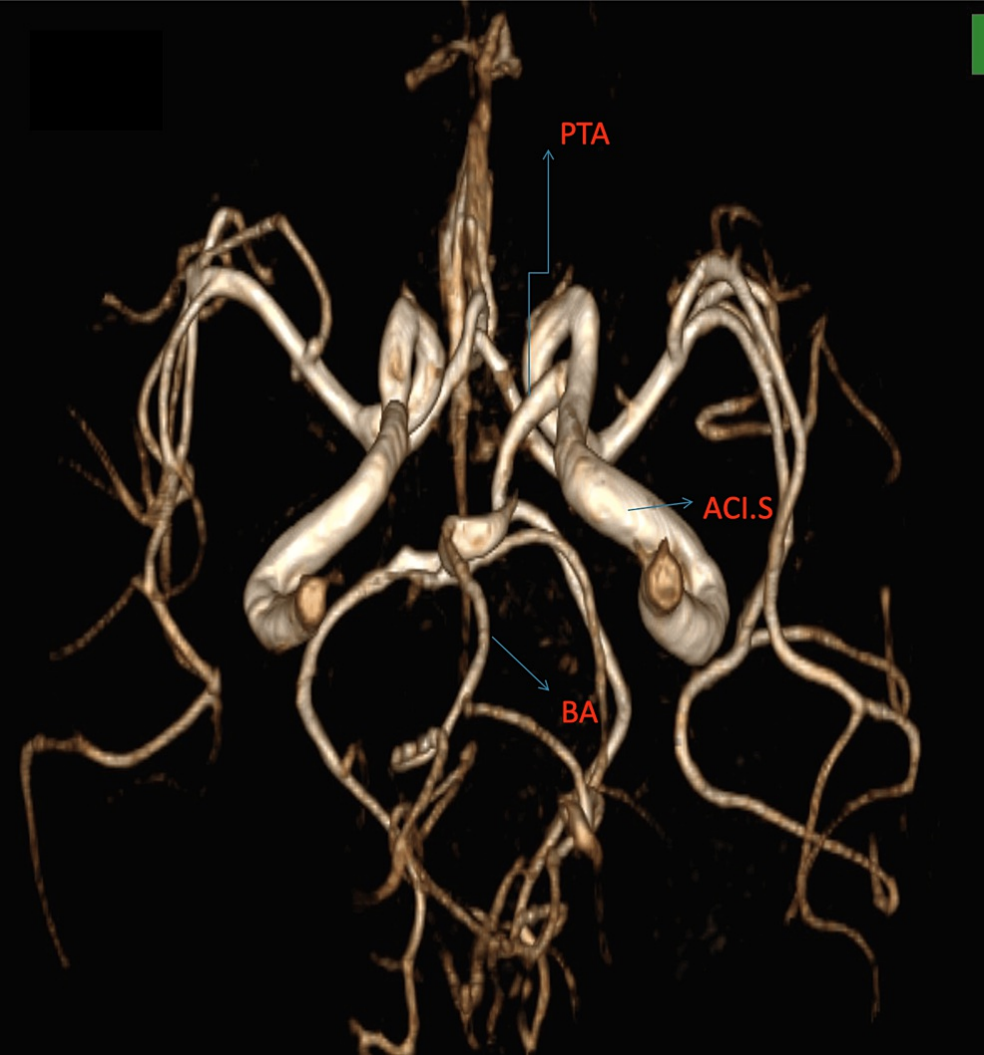
Top down view of the 3D reconstruction of the 3D TOF sequence PTA: persistent trigeminal artery, ACI.S: left internal carotid artery, BA: basilar artery

**Figure 3:**
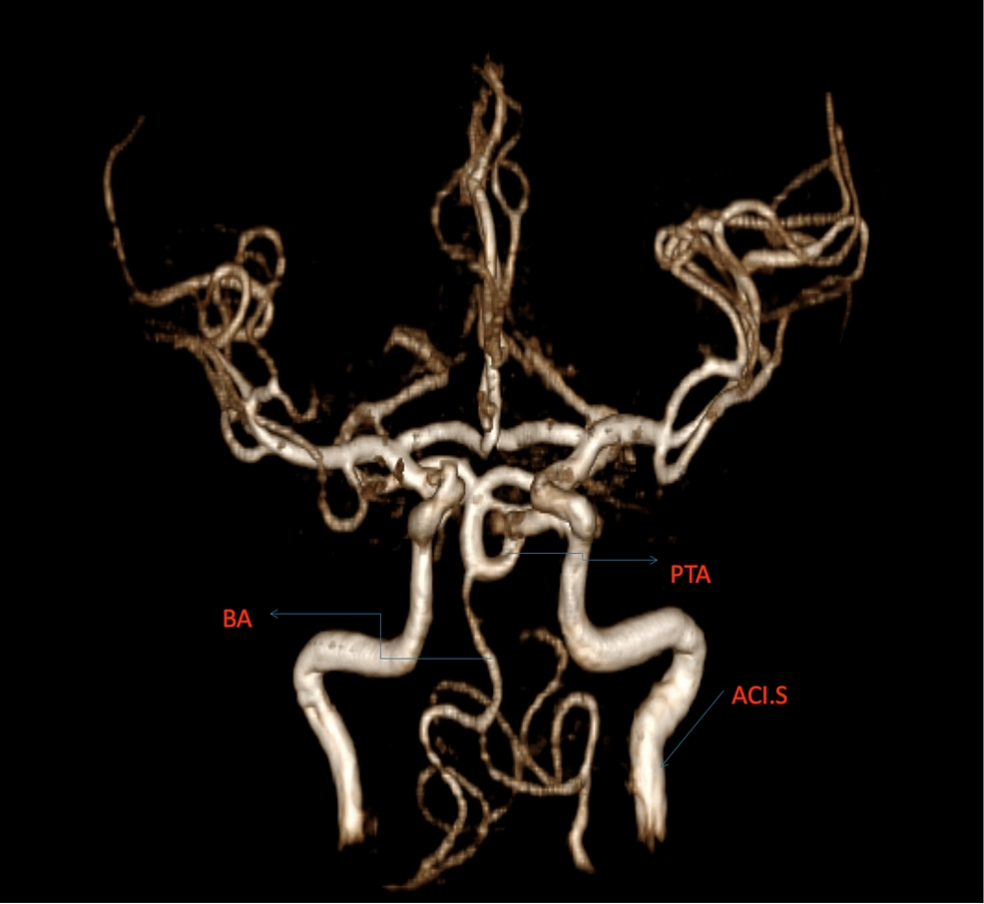
Front view of the 3D reconstruction of the 3D TOF sequence PTA: persistent trigeminal artery, ACI.S: left internal carotid artery, BA: basilar artery

**Figure 4:**
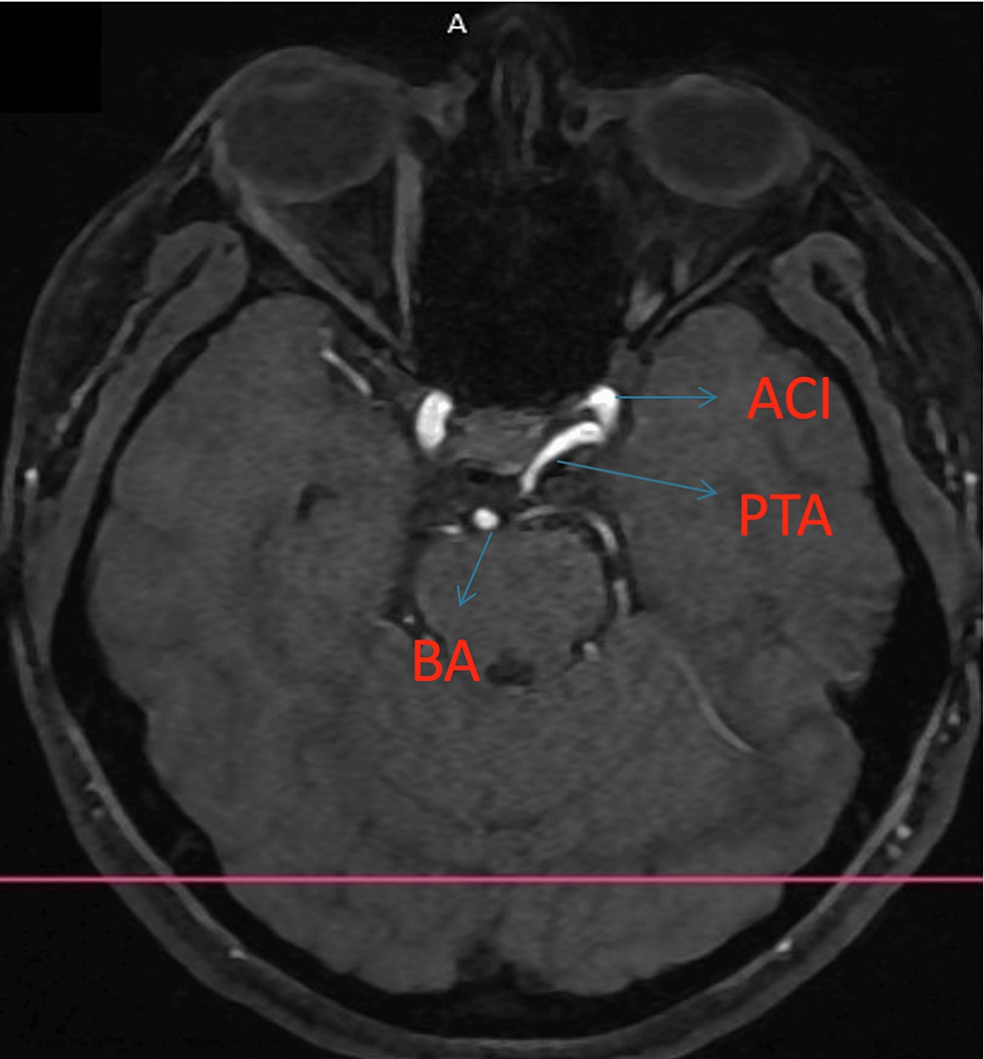
Axial view from the 3D TOF sequence PTA: persistent trigeminal artery, ACI: internal carotid artery, BA: basilar artery

**Figure 5:**
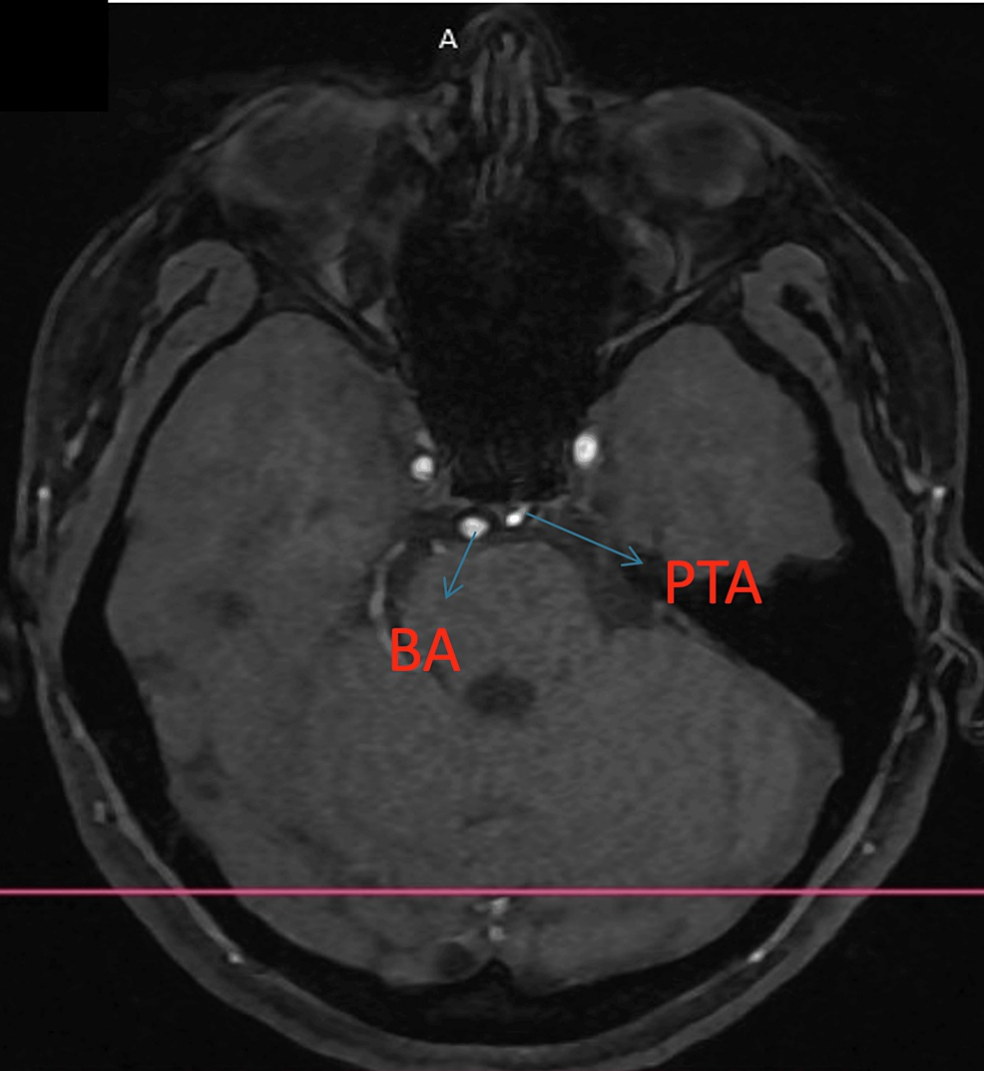
Axial view from the 3D TOF sequence PTA: persistent trigeminal artery, BA: basilar artery

Using the Saltzman classification, we classified the PTA as type I and the Salas classification as a medial type. Figures 1-3 (3D reconstruction of the 3D TOF sequence) and Figures 4-5 (axial view of the 3D TOF sequence) present the imagistic findings of the PTA in this patient. In Figure 4, we can see the "Tau sign,” which is a characteristic finding in a PTA case [7].

The patient did not want to continue the course of the investigations, so a classic angiography was not performed. While there was no brain aneurysm seen, there is a chance of the patient developing one in the future. The follow-up plan includes requesting further investigations in case the symptoms persist or new symptoms appear. Regular monitoring of the malformation to document if an aneurysm developed at this level is indicated in this case. As it is a Saltzman type I and the VA and BA are hypoplastic, complications of the anterior brain circulating system, such as artery dissection, atherosclerotic plaques, or a stroke, can cause, besides anterior circulation symptoms such as dysarthria, limb weakness, cognitive impairment, and posterior circulation symptoms such as ataxia, nystagmus, and vertigo.

## Discussion

Most of the trigeminal artery persistence cases are discovered incidentally, and most often, patients do not experience any symptoms [6]. If the PTA is symptomatic, it can cause symptoms like III, IV, or VI nerve palsy [6,13], trigeminal neuralgia, vertigo, ataxia, stroke, or migraines due to its course through the cavernous sinus and prepontine cistern, intersecting with the respective nerves [6,13-14]. A study by de Bondt et al. in 2006 found a 2.2% frequency of PTA in patients suffering from trigeminal neuralgia [15].

Paradoxically, a PTA can also lead to a posterior territory stroke with an embolic development that originates in the anterior territory, supplying arteries such as the ICA [15].

Supplying the posterior circulation can be a protective factor in the case of a stenosis of the supplying arteries of this territory. A case report describes a dissection of the vertebral artery and the protective collateral flow from the PTA, which caused the symptoms to be milder than usual [16]. Another case where this mechanism is useful is in the case of subclavian steal phenomena, when an occluded artery or a severe stenotic artery is perfused via a donor artery, reducing the blood flow to the respective territory [17]. In the case of a subclavian steal phenomenon, the posterior cerebral circulation is the donor, and having a connection with the anterior one reduces the hypoperfusion of this territory [6,18].

Another "advantage” of this malformation can be observed in patients who have stenosis in the carotid system; the reversal flow from the posterior brain circulation to the anterior one can keep hypoperfusion symptoms from appearing [8]. While this theory seems to be a protective factor for patients, there are very few studies that demonstrate this effect. Another advantage of this malformation is that it can be used as a pathway for endovascular intervention in the posterior territory, especially if the vertebrobasilar system is hypoplastic and cannot be used to deliver a stent [19].

If the PTA exists as the sole supply of the posterior circulation, in the case of a hypoplastic VA, reverse flow to the anterior circulation cannot occur, and a stenosis on the carotid system can lead to symptoms of vertebrobasilar insufficiency (VBI), the most common symptom being dizziness, but it can present with symptoms such as vertigo, ataxia, and double vision or loss of vision [20]. These clinical features can make a clinical diagnosis more difficult to make, requiring imagistic investigations to exclude other causes of the symptoms.

Another associated clinical condition consists of aneurysms. Davis et al. first reported it in 1956 [21]. In 1999, Cloft et al. reported a prevalence of 3% of brain aneurysms in a series of 34 patients, similar to the prevalence of brain aneurysms in the general population [22]. Other studies suggest a higher incidence of aneurysms in patients with PTA, approximately 13.8 to 27.8% [11]. Another study, conducted by O’uchi and O’uchi in 2010, assessing 16,415 MRAs, reported an incidence of 3.9% of cerebral aneurysms in the 103 patients with PTAs [4]. 

Symptoms of a PTA aneurysm (PTAA) can include cranial nerve palsy, usually III, IV, V, or VI, depending on the site of the aneurysm [6,22].

In the case of a ruptured PTA aneurysm, patients usually present with symptoms such as headache and posterior cranial fossa symptoms; sometimes cranial neuropathy and facial pain can be observed in the case of a large or giant cavernous PTAA [3,22].

Treatment of an aneurysm of this type can be by endovascular means, such as coiling or stent-assisted coiling, or by surgical means, such as microsurgical clipping. The surgical approach is considered more difficult because of its deep positioning and close proximity to the cranial nerves [19,23].

Another associated disease related to PTA is a trigeminal cavernous fistula, which represents a direct communication between the PTA and the cavernous sinus [6]. This presents with ocular symptoms such as ophthalmoplegia and pulsatile exophthalmos [6]. Another complication of this fistula is having high pressure in the cerebral venous system, which can lead to intracranial hemorrhages [3,6]. Other studies suggest that there are also other vascular anomalies related to PTA, such as arterial-venous malformations and MoyaMoya disease; in the case of MoyaMoya disease, there is an observed incidence ten times higher than in a normal population [24].

In the case of a transcellular PTA, documenting it before an endoscopic surgery for pituitary adenoma is very important to avoid sectioning it during surgery, which can cause a massive bleed. According to the Salas classification, this is usually associated with a medial type, but lateral types need to be taken into account in preoperative planning [25-26]. Medial-type PTA was also reported to cause hyperprolactinemia due to the stalk effect [27].

In our case, the patient only had a persistent headache, while no other aneurysms were present. These symptoms are very unspecific for PTA, but having a hypoplastic posterior circulation can lead to posterior circulation hypoperfusion symptoms such as dizziness, ataxia, or vision problems [6,8,20], in the case of carotid atherosclerotic plaques or other conditions that restrict the anterior brain circulation blood flow [8]. Furthermore, reverse flow is not very likely to happen because of the anatomy of the posterior brain circulation in this patient. The patient should consider having a healthier diet and reducing the risk factors for atherosclerosis because of this malformation [8,28]. Another take-home point for this patient is the need for regular checks on the state of the malformation, assessing the appearance of an aneurysm before it causes symptoms or rupture.

## Conclusions

The persistence of the trigeminal artery is the most common embryonic vascular persistence of carotid vertebrobasilar anastomoses. The diagnosis is usually incidental, following an angioCT or an MRA, in patients with no symptoms or non-specific symptoms such as headaches, as it is in our case. Usually, the PTA is associated with other aneurysms of the brain arteries, and even if they are not present at the time of the examination, they can appear over time. Imagistic investigations of the brain arteries are important in patients with persistent headaches as they can discover malformations such as PTA, which can prevent other more serious complications such as aneurysms, cavernous fistulas, or nerve palsy.

While most of the PTA symptoms are non-specific, they should be considered a differentiated diagnostic because of the broad range of symptoms. AngioCT and MRA are non-invasive and easily accessible investigations that can diagnose this malformation.
